# Successive Increases in the Resistance of *Drosophila* to Viral Infection through a Transposon Insertion Followed by a Duplication

**DOI:** 10.1371/journal.pgen.1002337

**Published:** 2011-10-20

**Authors:** Michael M. Magwire, Florian Bayer, Claire L. Webster, Chuan Cao, Francis M. Jiggins

**Affiliations:** 1 Department of Genetics, University of Cambridge, Cambridge, United Kingdom; 2 Institute of Evolutionary Biology, University of Edinburgh, Edinburgh, United Kingdom; University of California Davis, United States of America

## Abstract

To understand the molecular basis of how hosts evolve resistance to their parasites, we have investigated the genes that cause variation in the susceptibility of *Drosophila melanogaster* to viral infection. Using a host-specific pathogen of *D. melanogaster* called the sigma virus (Rhabdoviridae), we mapped a major-effect polymorphism to a region containing two paralogous genes called *CHKov1* and *CHKov2*. In a panel of inbred fly lines, we found that a transposable element insertion in the protein coding sequence of *CHKov1* is associated with increased resistance to infection. Previous research has shown that this insertion results in a truncated messenger RNA that encodes a far shorter protein than the susceptible allele. This resistant allele has rapidly increased in frequency under directional selection and is now the commonest form of the gene in natural populations. Using genetic mapping and site-specific recombination, we identified a third genotype with considerably greater resistance that is currently rare in the wild. In these flies there have been two duplications, resulting in three copies of both the truncated allele of *CHKov1* and *CHKov2* (one of which is also truncated). Remarkably, the truncated allele of *CHKov1* has previously been found to confer resistance to organophosphate insecticides. As estimates of the age of this allele predate the use of insecticides, it is likely that this allele initially functioned as a defence against viruses and fortuitously “pre-adapted” flies to insecticides. These results demonstrate that strong selection by parasites for increased host resistance can result in major genetic changes and rapid shifts in allele frequencies; and, contrary to the prevailing view that resistance to pathogens can be a costly trait to evolve, the pleiotropic effects of these changes can have unexpected benefits.

## Author Summary

Though much is known about host–parasite coevolution in plants, relatively little is understood in animals. Most studies using animal systems have focused on either generalist parasites or those that do not naturally occur in the host. The sigma virus is specific to *Drosophila melanogaster*, which provides the unique opportunity to study natural coevolution in a well-established model organism. In order to gain a better understanding of host–parasite coevolution, we have set out to identify novel viral resistance genes using the sigma-*Drosophila* system. Here we identify two successive mutations that provide increasing resistance to the sigma virus. The first of these, a transposable element insertion within a gene called *CHKov1*, is already known to provide resistance to insecticides. There is evidence that the novel gene product resulting from this insertion has been under positive selection pressure long before the use of pesticides. Two duplications of this gene region have resulted in further resistance to sigma virus. We believe that selection for resistance to the sigma virus led to the added benefit of resistance to insecticides.

## Introduction

The presence of a parasite elicits strong selection pressures for the host to evolve increased resistance and the parasite to overcome host defences. This can drive rapid changes in allele frequencies in both organisms and result in “Red Queen” evolution, where both species must constantly evolve just to maintain a fitness status quo [1]. Generation times, population sizes, mutation rates and migration rates all affect the evolutionary potential of hosts and parasites, and these factors mean that in many cases the parasite will be evolving faster than the host [2]. Therefore the host is under constant selection to evolve new forms of resistance to the parasite, and this makes host resistance an excellent model to study the evolution of adaptation.

Identifying the genes underlying the evolution of resistance can provide insights into this process, revealing the types of mutation involved, the nature of selection acting on resistance, and the molecular mechanisms involved in evolving resistance to infection. A substantial amount of work has been done to study the genetics of host-parasite co-evolution in plants, and we have a broad knowledge of plant resistance (*R*) gene genetics [3]. Unfortunately this is not true for the animal kingdom, especially invertebrates. Aside from a handful of studies on disease vectors, much of the work on invertebrates tends to be purely phenotypic or has not been done with naturally co-evolving systems. Identifying the genes causing variation in the resistance of invertebrates to viruses will allow us to get at many of the mechanisms underlying the evolution of resistance and provide insights to the nature of co-evolution.

The antiviral immune defences of *Drosophila* have been the target of much research in recent years, with RNAi, autophagy and other pathways proving to be important [4]–[7]. However, on an evolutionary timescale, changes to the immune system are not the only way in which hosts can defend themselves against viruses. Several insects, including *Drosophila melanogaster*, have developed a symbiosis with the bacterium *Wolbachia* that provides resistance to a range of RNA viruses [8]–[11]. Viruses also rely on the host cellular machinery for all stages of their replication cycle, and changes to these host factors may also lead to the evolution of resistance, for example by blocking entry in host cells [12].

The discovery of genes causing variation in resistance can also allow us to infer the selection pressures acting on host alleles during co-evolution [3], [13]. Co-evolution can result in two main forms of selection: new resistance alleles may continually arise by mutation and be fixed by directional selection, or negative frequency-dependent selection can maintain polymorphisms of resistant and susceptible alleles [1], [13]–[14]. To complicate matters, selection pressures on host alleles can be very dynamic, not only depending on allele frequencies in the parasite [15], but also on changing environmental conditions. It is also of interest to understand the genetic architecture of resistance and the nature of the mutations involved. For example, is the resistance level primarily controlled by alleles of small or large effect, and is it the result of regulatory or coding changes or both? By addressing all of these questions, the identification of host genes experiencing strong selection will therefore help to develop better models of co-evolution.

We have investigated the genetics of resistance to the sigma virus, the only naturally occurring host-specific parasite known in *D. melanogaster* [16]–[17]. Host specificity is important, as when a parasite infects a single host species there is particularly strong selection for reciprocal adaptation, and such “tight” co-evolution simplifies the arduous task of understanding how co-evolution operates. The sigma virus is a member of the rhabdovirus family, and has a negative-sense RNA genome [18]. It is only transmitted vertically from parent to offspring [18]. In this study we have investigated a resistance gene called *ref(3)D*, which had previously been mapped between two visible markers on the right arm of the 3^rd^ chromosome [19].

## Results

### Genetic mapping

**Figure 1:**
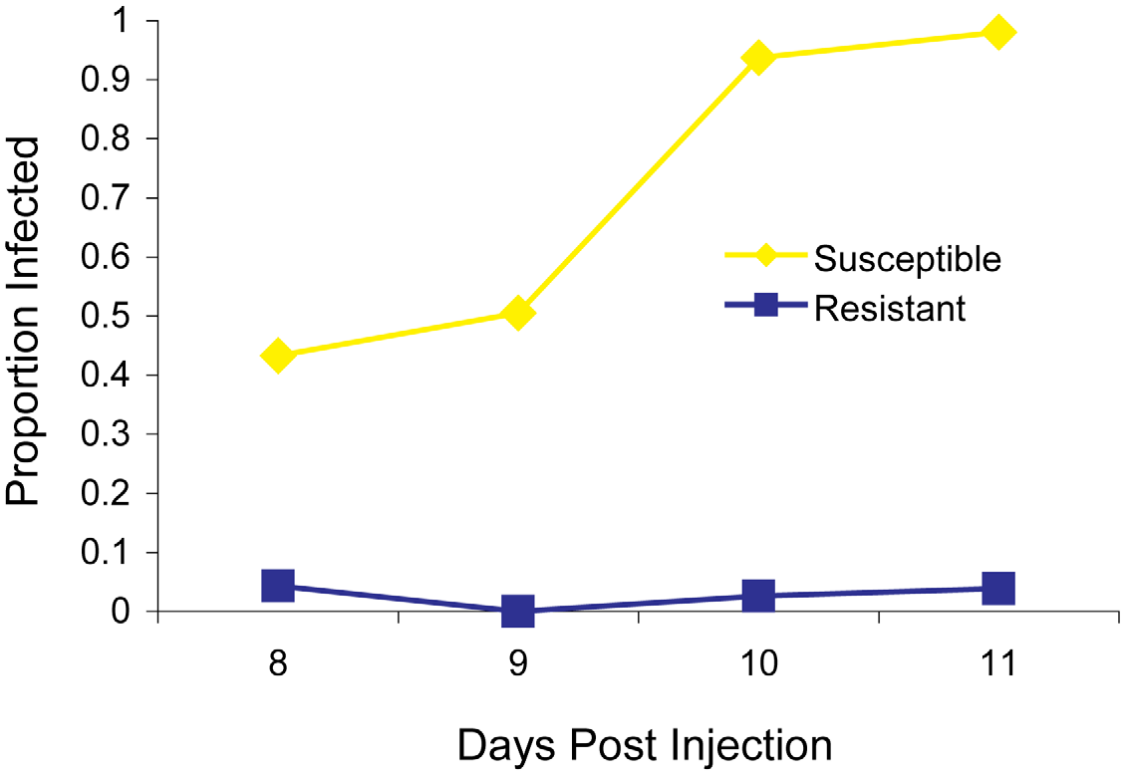
The infection rate of resistant and susceptible flies. A total of 297 flies from the parental fly lines (susceptible 22a and resistant OOP) were injected with the sigma virus and tested for infection with our CO_2_ assay 8–11 days later.

The two fly lines that we began our experiments with differed dramatically in their resistance to the sigma virus —11 days after injection less than 5% of the flies from the resistant OOP line showed the symptom of being paralysed by CO_2_, compared to over 95% of flies from the susceptible 22a line (Figure 1). Previous work has mapped a gene called *ref(3)D*, which affects sigma virus replication, to the third chromosome of this fly stock [19]. However, it also contains a gene with an allelic variant that reduces transmission of the sigma virus through sperm, so we first removed this allele to avoid complications in identifying *ref(3)D*. This was accomplished by crossing OOP and 22a to generate a line with a recombination event between the suspected locations of each gene (92–94 cM region). The resulting line was homozygous for both the resistant allele of *ref(3)D* and the allele of the other gene that results in high rates of transmission through sperm, and this was used in subsequent experiments.

**Figure 2:**
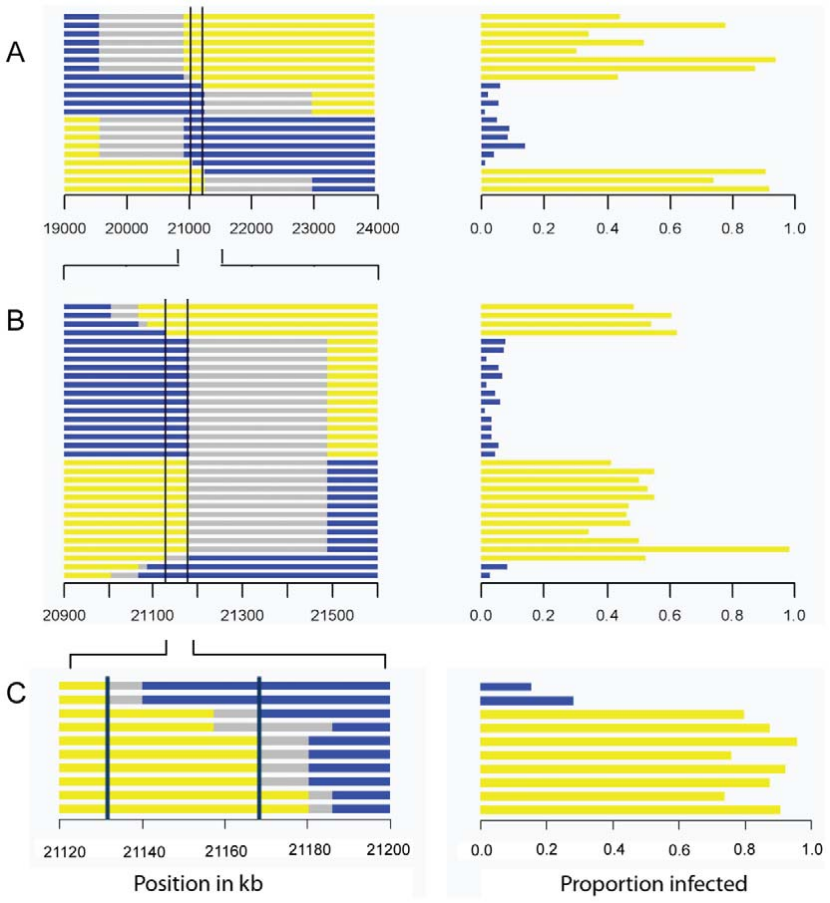
Mapping resistance to the sigma virus. The left hand panel shows the genotype of the flies inferred from molecular markers, with each horizontal bar representing the chromosome of a different homozygous recombinant fly line. The blue region is derived from the resistant parent, yellow from the susceptible parent, and grey is not determined. The scale represents the position in kb in release 5.31 of the *Drosophila* genome. The right hand panel shows the proportion of flies that were infected in our experiment. There is a perfect association between genotype and phenotype in the region between the two vertical bars. As described in the text, we repeated the experiment three times, each time selecting lines that had recombined within the region identified by the previous experiment. This allowed us to map the gene to a 192 kB region in Experiment A, a 60 kb region in experiment B, and a 36 kB region in experiment C.

To map *ref(3)D*, we produced lines that carried a homozygous third chromosome that was a recombinant between the resistant and susceptible stocks. We used molecular markers to screen 191 recombinant flies to identify those that had recombined in a 12 cM interval believed to contain the gene, and created 21 homozygous recombinant lines in this anticipated region. These lines were injected with the sigma virus and genotyped with molecular markers across the region (Figure 2A). There was a clearly bimodal distribution of infection rates, with some lines being highly resistant and others highly susceptible. Furthermore, there was a perfect association between infection rates and genotype across a 182 kb region (Figure 2A; Wilcoxon Rank Sum Test: *W* = 110, *P* = 1.2×10^−4^).

This process was repeated to generate recombinants in the 2 cM interval that contains the resistance gene. This time we screened 1920 flies for informative recombinants and 32 new homozygous recombinant lines were generated in this new region. Again, after injecting the virus these could be clearly categorized into resistant and susceptible lines. After genotyping the lines, this experiment reduced the region where there is a perfect association between genotype and phenotype to 60 kb (Figure 2B; Wilcoxon Rank Sum Test: *W* = 256, *P* = 1.5×10^−6^).

To select for recombinants in this smaller region we used phenotypic markers rather than molecular markers. We combined two *P*-elements carrying eye-color markers to produce a susceptible mapping stock (2GT1), crossed this to a resistant fly line, and selected recombinants that carried just one of the two markers. Using this approach we generated 10 lines that were homozygous for the recombinant chromosome. As before, these lines were assayed for resistance to the sigma virus and genotyped for several markers across the 60 kb candidate region. This reduced the region that could contain the gene to 36 kb (Figure 2C; Wilcoxon Rank Sum Test: *W* = 16, *P* = 0.04).

**Figure 3:**
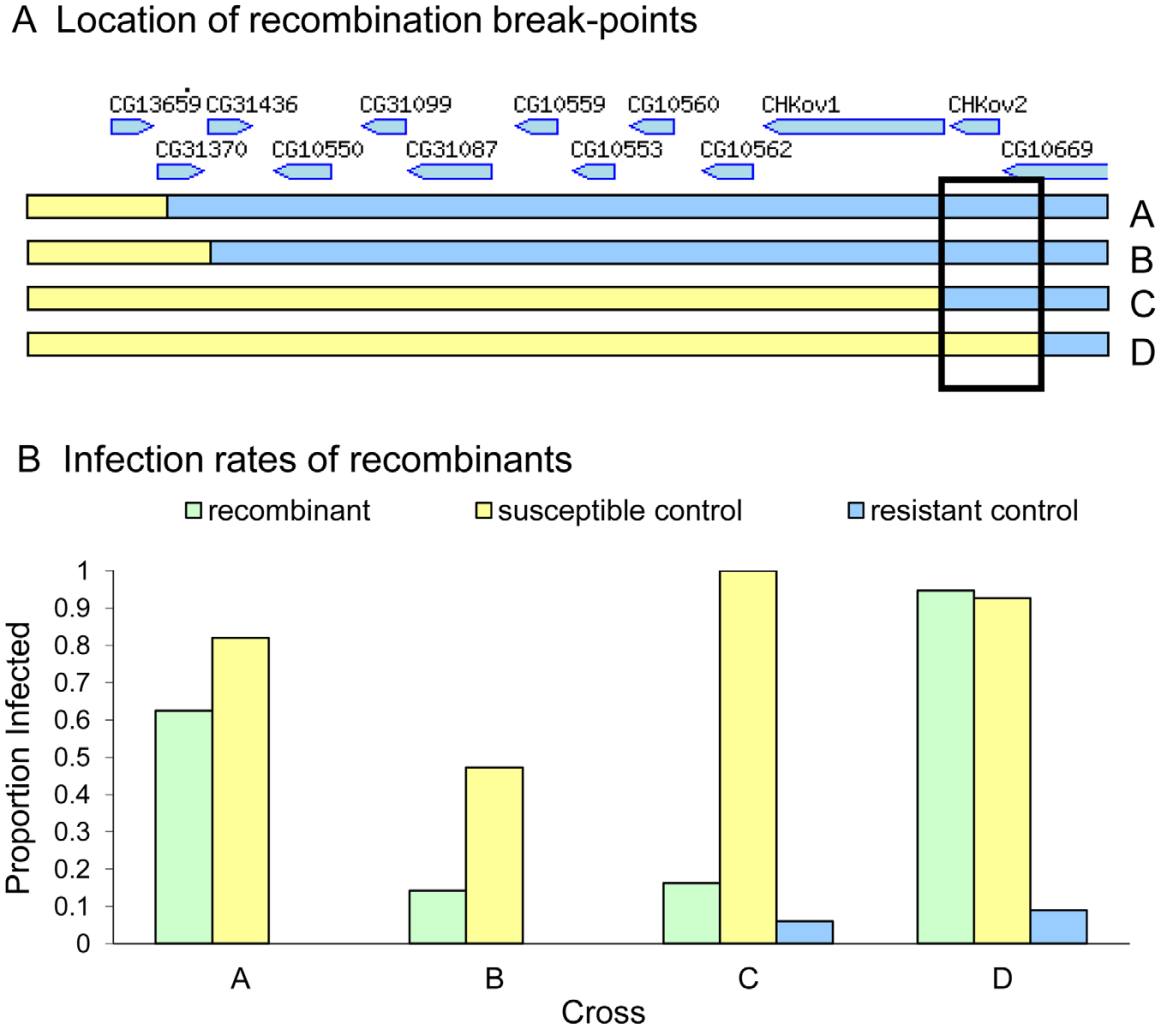
Mapping resistance using site-specific male-induced recombination. Recombination was induced at the four positions shown in panel A to produce four recombinant lines (A, B, C and D). Relative gene locations in the 36 kb region are indicated [36]. The susceptibility of these lines to the sigma virus is shown in Panel B (green bars). There is a large difference in the susceptibility of recombinants C and D, suggesting that the causative gene is located in the region shown in the box. The infection rate of susceptible controls is shown in yellow and the resistant controls in blue. In crosses A and B we were unable to create any resistant controls, and are therefore unable to control for other genes in the genetic background of these stocks.

To map the gene within in this region, we induced site-specific recombination in males using *P*-elements. In this experiment we crossed transposable element lines that were susceptible to the sigma virus (data not shown) to a resistant line, and induced recombination at the location of the *P*-element. We successfully produced four recombinants that were viable as homozygotes. To control for the effects of genetic background, lines that lacked a recombination event were also generated using the same crossing scheme, so they either had the susceptible chromosome containing the transposable element or the resistant chromosome. To check that recombination had occurred, we scored molecular markers flanking the transposable element positions in each line. We injected the recombinant lines and respective controls with the sigma virus (Figure 3), and found that there was a striking difference between the resistance of recombinants between two sites located just 3089 bases apart in the published genome (3R:21155073..21158162, *D. melanogaster* genome version 5.31.). This region contains all of *CHKov2* plus the 3′ end of *CG10669* in the published genome sequence (part of the fifth exon, all of the sixth exon and the 3′UTR).

### Sequencing and gene expression

**Figure 4:**
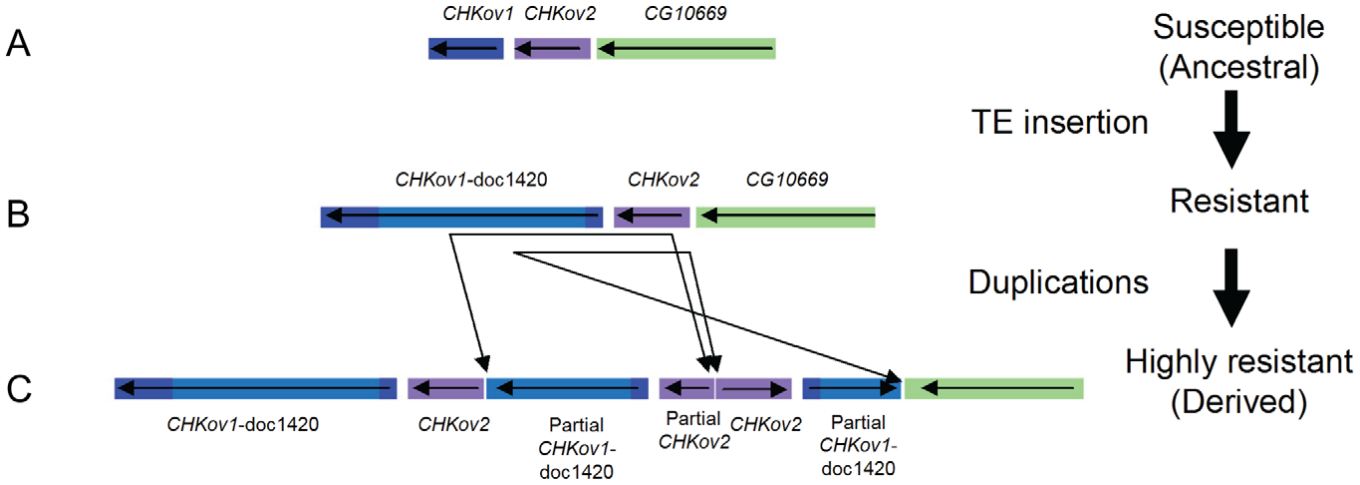
The evolution of *CHKov1* and *CHKov2*. This region has undergone two changes that have each increased resistance to the sigma virus. First, a transposable element inserted into *CHKov1*, truncating the coding sequence of the gene. Second, this truncated gene was duplicated twice. The duplication also resulted in two complete and one partial copy of *CHKov2*.

To identify the polymorphisms that could be causing resistance, we sequenced the region around *CHKov2* and found that there had been a complex rearrangement in the resistant line (Figure 4C, highly resistant line). The susceptible line had a gene order that is the same as the published *Drosophila* genome (Figure 4B, note that this is described as ‘resistant’ in the figure as a more susceptible allele is described below). As is the case in the published genome sequence, in both our resistant and susceptible lines a naturally occurring *Doc* transposable element has inserted into the protein coding region of *CHKov1*, which is a paralog and neighbour of *CHKov2*. Previous research has shown that this insertion results in two short transcripts being produced, which are predicted to encode truncated proteins [20]. However, in the resistant line there are two duplications, both of which involve partial sequences of both *CHKov1* and *CHKov2*. The first duplication includes a large portion of the 5′ end of *CHKov1* (including some upstream intergenic sequence) and approximately two-thirds of the 3′ end of *CHKov2*. The second duplication is in the reverse orientation, and includes all of *CHKov2* and the 5′ end of *CHKov1* (compared to the first duplication, this includes less of the *Doc* element insertion, exactly the same protein coding region and an identical region of the upstream intergenic sequence). It is highly likely that this rearrangement is causing the difference in resistance, as in the region mapped by male recombination there is only one single nucleotide polymorphism (SNP) outside of the rearrangement that differs between the resistant and susceptible lines.

This rearrangement could confer resistance to viruses either by altering the expression of the genes involved, or due to coding changes (the only coding sequence which is altered is the truncation of one of the duplicates of *CHKov2*). We therefore used quantitative rtPCR to examine whether the expression of *CHKov1* or *CHKov2* is different in the resistant and susceptible flies. It has previously been shown that neither of the CHKov genes change expression after injection with sigma [21], so any novel changes in expression could be attributed to the rearrangement. Six days after injection with the sigma virus *CHKov2* expression was 5.6-fold greater in the resistant lines than the susceptible lines (Wilcoxon Rank Sum Test: *W* = 86, *P* = 0.0001) and 12 days after injection it was 9.6-fold greater (Wilcoxon Rank Sum Test: *W* = 88, *P* = 2.6×10^−5^). In contrast there was no evidence for a change in the expression of *CHKov1*, despite this gene being amplified to three copies in the resistant line (1.9 fold greater expression in susceptible lines on day 6, Wilcoxon Rank Sum Test: *W* = 24, *P* = 0.11; 1.4 fold greater expression in susceptible lines on day 12, Wilcoxon Rank Sum Test: *W* = 29, *P* = 0.24).

### Viral load

In the experiments above we have used a symptom of infection — paralysis on exposure to CO_2_ — to test if flies are infected. To check whether the resistance gene is reducing viral titres rather than simply altering CO_2_ sensitivity itself, we used quantitative PCR to estimate the relative copy number of the viral genome in resistant and susceptible flies. Using the same samples that we used to examine gene expression, we found that there was an approximately 79–fold decrease in sigma virus load in resistant lines 6 days after the virus was injected (Wilcoxon Rank Sum Test: *W* = 0, *P* = 3×10^−5^) and a 138–fold decrease after 12 days (Wilcoxon Rank Sum Test: *W* = 0, *P* = 3×10^−5^).

### Genetic variation in a natural population

As the rearrangement of the *CHKov1* and *CHKov2* genes that confers resistance to the sigma virus was originally found in a natural population in Europe, we examined its frequency in nature. To do this we used Freeze 1 of the *Drosophila* Genetic Reference Panel (DGRP) (http://www.hgsc.bcm.tmc.edu/project-species-i-Drosophila_genRefPanel.hgsc), which is a set of highly inbred North American fly lines whose genomes have been sequenced. As the genome sequences were produced from short-read data, rearrangements and transposable element insertions are not reliably assembled. We therefore used PCR to genotype all the lines for both the *Doc* element in *CHKov1* and the complex rearrangement. The *Doc* insertion was present in most of the lines (155 were homozygous for the insertion, 29 were homozygous without it, and 8 were heterozygous, likely due to insufficient inbreeding.), but the rearrangement was not found in any of the 192 lines tested. Therefore this rearrangement is not an important cause of virus resistance in this population.

As the truncated version of *CHKov1* has been duplicated in the most resistant allele (Figure 4C), we tested whether the *Doc* element insertion in *CHKov1* was itself associated with resistance. We injected 11870 flies from 186 of the DGRP lines with the sigma virus and tested them for infection with the CO_2_ assay 13 days later. We found that the insertion is associated with a highly significant drop in infection rates (Bayesian generalised linear mixed model: *P*<0.001). Using this statistical model, we estimate that the *Doc* insertion is associated with a 52% drop in infection rates from 82% to 30% (95% C.I. on drop: 42%–64%). It should be noted that the susceptible line used in the mapping experiment above contains the *Doc* insertion. Therefore the three alleles in this region shown in Figure 4 have a hierarchy of resistance, with the ‘rearranged’ allele being most resistant (Figure 4C) and the *Doc* insertion having intermediate resistance (Figure 4B). The sequence in *Drosophila simulans* has neither the *Doc* insertion nor the rearrangement, indicating that the most susceptible allele is the ancestral state (Figure 4A), the allele of intermediate resistance arose next following the *Doc* insertion, and then a rearrangement occurred that lead to a further increase in resistance.

**Figure 5:**
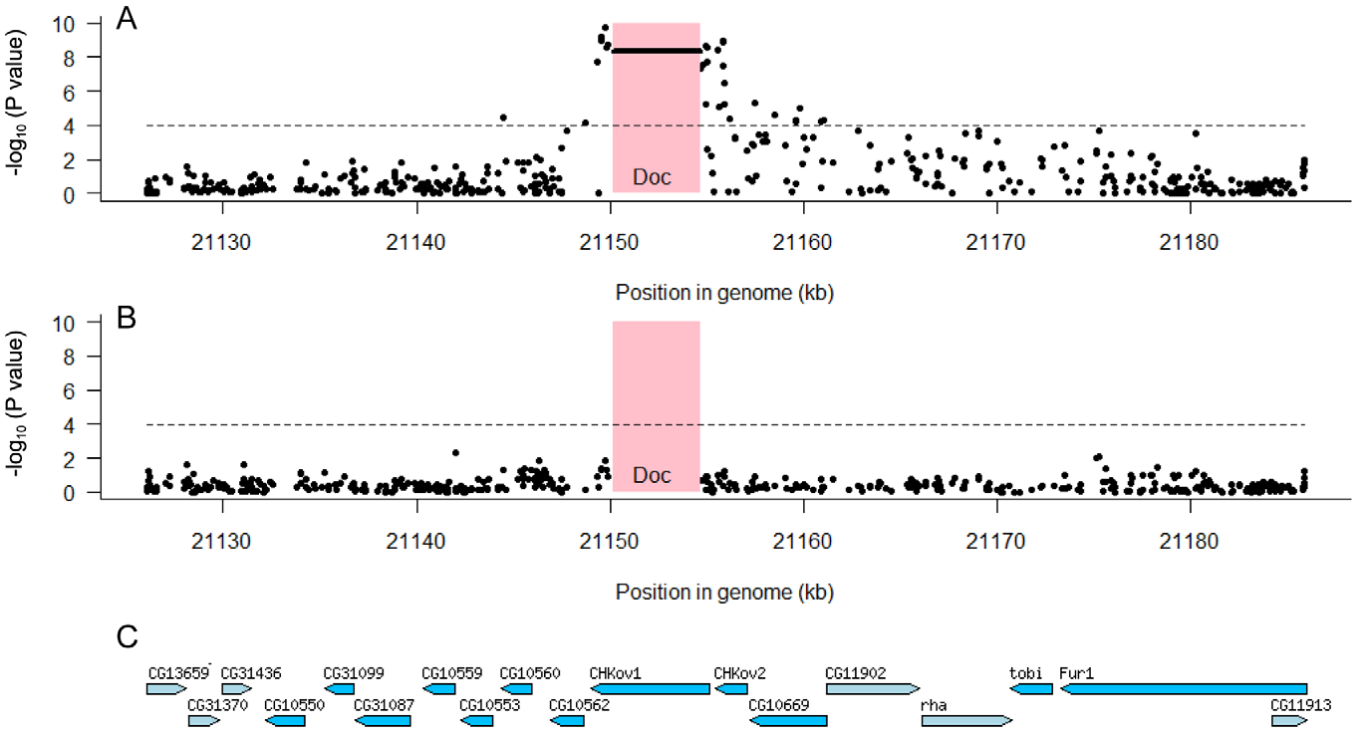
Associations between SNPs in the 60 kb region around the *CHKov* genes and resistance to the sigma virus. We tested for associations between SNPs and resistance using 150 highly inbred fly lines whose genomes have been sequenced. The pink box shows the position of the *Doc* transposable element insertion and the dashed line is the significance threshold after Bonferroni correction. Panel A shows that both the *Doc* insertion (horizontal line in pink box) and a large number of SNPs to either side have highly significant associations. Panel B shows that these associations disappear when the *Doc* element is included as an explanatory factor in the analysis, which indicates that all the significant SNPs are in linkage disequilibrium with the *Doc* insertion. Panel C shows the locations of genes in this region [36].

To examine whether any other polymorphisms in this region are associated with resistance we used the data from the genome sequences of the DGRP lines. Using 150 lines whose genomes have been sequenced we examined the 60 kb region which we mapped in our first set of experiments (Figure 2B). In the regions flanking *CHKov1* we found that 32 of 468 SNPs in the region were significantly associated with resistance to the sigma virus after Bonferroni correction (Figure 5A). However, there is extensive linkage disequilibrium between the *Doc* insertion and surrounding sites (see below; [20]), so all of these associations could all be caused the same polymorphism. We therefore repeated the analysis, but this time included the presence or absence of the *Doc* insertion in the model. We found that none of the associations were significant (Figure 5B), so the most parsimonious interpretation is that a single polymorphism in this region is causing resistance. As the *Doc* insertion has such a dramatic effect on the protein encoded by *CHKov1*, this is most likely to be the cause of resistance.

The mapping data together with these association studies therefore provide strong evidence that there are two different polymorphisms in this region that make flies resistant involving the *Doc* insertion and its subsequent duplication. However, we still wished to confirm that none of the other SNPs associated with resistance in the DGRP lines could contribute to the difference between the resistant and susceptible lines used in the mapping experiments. We therefore sequenced this entire 60 kb region from both the resistant and susceptible lines (OOP and 22a), and identified 191 SNPs and 11 indels that differed between these lines and were present in the DGRP genomes. None of these polymorphisms were significantly associated with resistance to sigma (after corrections for multiple testing; Figure 5B), and only two of them fell within a 30 kB region around the duplication implicated in resistance. This confirms that different genetic changes are affecting resistance in the DGRP lines and causing the difference between the two lines we used in the mapping experiments.

### Genetic diversity around CHKov1

**Figure 6:**
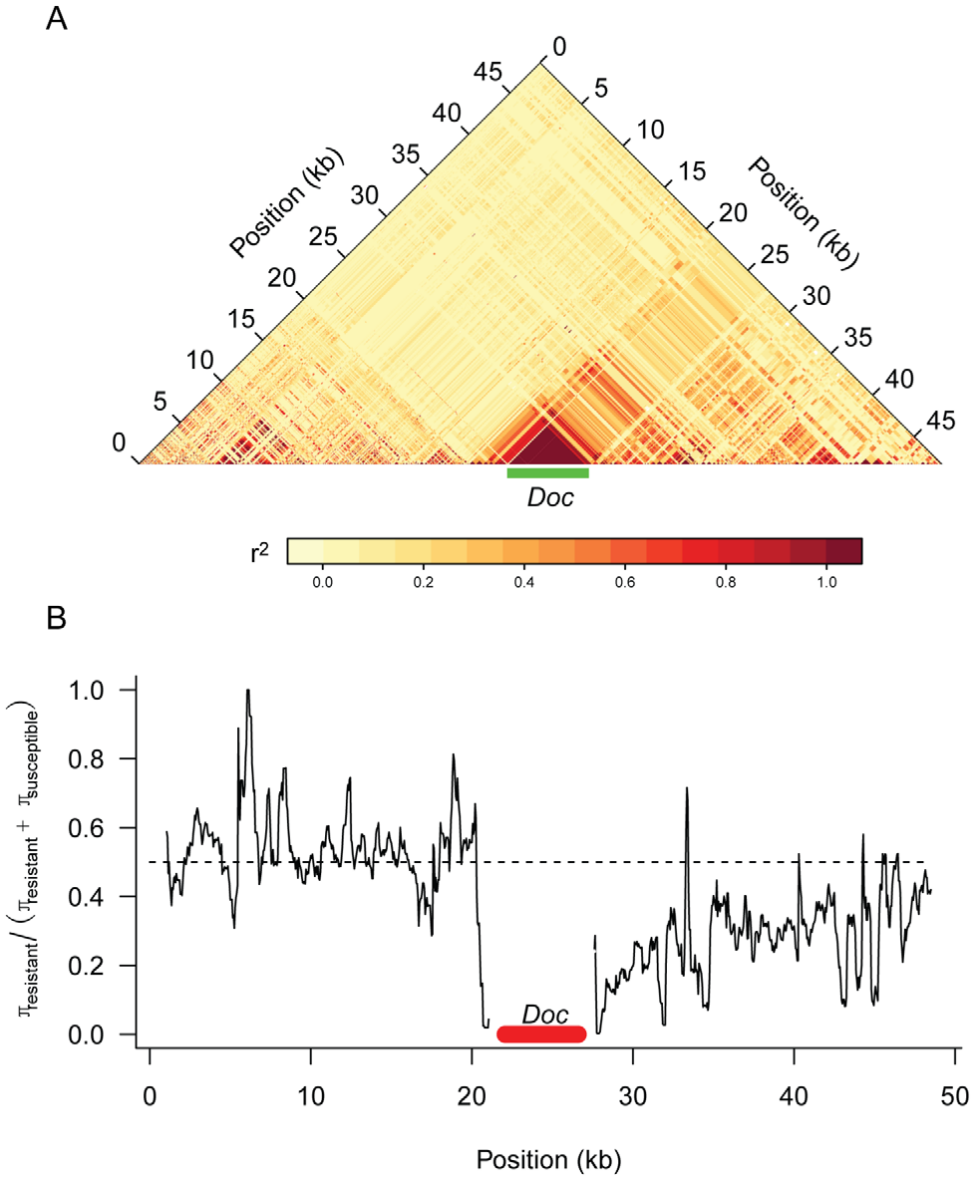
Genetic variation around the *CHKov* genes. Panel A shows linkage disequilibrium (*r^2^*) between pairs of sites in the DGRP sequences. Only polymorphisms where the minor allele occurred in 5 or more lines are shown. Panel B shows a sliding window plot of the ratio of genetic diversity (π) among the resistant alleles to the sum of the genetic diversity of the susceptible and resistant alleles. The dotted line is the expected value if the two classes of alleles had the same genetic diversity. A window size of 500 bp was used.

Previous studies have examined the pattern of genetic variation around the *Doc* insertion in *CHKov1* [20], but the sequences of all 192 DGRP lines provides us with a more complete dataset. We found that there is extensive linkage disequilibrium between the *Doc* insertion and surrounding sites that extends at least 25 kB to the 3′ end of the gene and a much shorter distance in the 5′ direction (Figure 6). In the region where sites are in linkage disequilibrium with the *Doc* insertion, there is greater genetic variation among the susceptible chromosomes than the resistant chromosomes (Figure 6), despite the resistant allele being most common. These observations are consistent with the conclusion of Aminetzach *et al* [20] that the *Doc* insertion has recently increased in frequency under directional selection.

## Discussion

We have found that two events have led to successive increases in resistance to the sigma virus (Figure 4). The first of these is a *Doc* transposable element insertion into the coding sequence of *CHKov1*. The second is a complex rearrangement that results in two duplications of *CHKov1* and the *Doc* element, further increasing resistance to sigma. As infection with the sigma virus reduces the fitness of infected flies [22], it is likely that selection for resistance to this common pathogen has led to the major structural changes in this gene and large shifts in resistance to the sigma virus. The first of these events, involving the insertion of the *Doc* element, caused the infection rate in our experiments to drop from 82% in flies with the susceptible allele to 30% in flies with the insertion. Transposable element insertions are known to be important in causing a number of major-effect mutations that are important in adaptations such as insecticide resistance [23]–[24]. In contrast to most of these changes, which tend to affect the regulatory regions upstream of genes [23], this *Doc* element has inserted into an exon and is expected to cause major changes to the structure of the protein. In its ancestral state, *CHKov1* is comprised of four exons that produce a single transcript. Previous research has shown that, by interrupting the original transcript, this *Doc* insertion results in two derived transcripts being produced, each of which contains both *Doc* element sequence and *CHKov1* sequence [20]. Assuming these transcripts are translated, this is likely to result in the protein losing its original enzymatic function, as neither of the new transcripts include the two protein domains encoded by the original transcript (a choline kinase domain and the PFAM domain DUF227) [20].

The second event to occur was a complex rearrangement of this region, which resulted in an even greater increase in resistance to the sigma virus than the original *Doc* insertion. The rearrangement leaves the fly with two full copies of *CHKov2*, a partial copy of *CHKov2*, and three full copies of the first derived transcript of *CHKov1* caused by insertion of the *Doc* element (Figure 4). The simplest explanation of how this rearrangement increases resistance is that the amplification of the region coding for the first derived transcript of *CHKov1* increases the expression of this new gene, and this in turn increases resistance. However, we were unable to find any evidence for the expression of *CHKov1* changing, suggesting that this is not the case. Furthermore, the coding region of *CHKov1* is unaffected by the rearrangement. However, the rearrangement is associated with a 6- to 9-fold increase in expression of *CHKov2*, suggesting that this may be the cause of resistance. *CHKov2* is a paralog of *CHKov1* which also has a predicted choline kinase activity [20], so it is possible that the two genes could both have antiviral effects through a similar mechanism.

These complex, sequential modifications to the *CHKov1* region are similar to a series of alleles of the gene *Cyp6g1* which increase resistance to the pesticide DDT [25]. In the case of *Cyp6g1*, successive increases in resistance to DDT were caused by the insertion of an *Accord* transposable element into the promoter followed by a gene duplication event and the insertion of an *HMS-Beagle* transposable element and a partial *P*-element [25]. Together with our results, this suggests that both transposable element insertions and gene-duplications can be important sources of major-effect mutations that contribute to phenotypic evolution.

It is well known that genes that increase resistance to pathogens often have pleiotropic effects on other components of fitness. For example, in *Drosophila*, selection for increased resistance to parasitoid wasps results in a decrease in competitive ability [26] and flies that are resistant to bacteria have reduced fecundity [27]. As these pleiotropic effects tend to be harmful, it is commonly thought that resistance to pathogens is a costly trait to evolve, and these costs are assumed in many theoretical models of coevolution [1]. However, previous research has found that the *Doc* element insertion in *CHKov1* increases resistance to organophosphate insecticides [20]. Therefore, contrary to received wisdom, this pleiotropic effect of this antiviral resistance allele would appear to be beneficial to the fly.

Although *CHKov1* is involved in pesticide and viral resistance, the molecular basis of these effects are not clear. Neither *CHKov1* nor *CHKov2* appear to be part of an induced response to the sigma virus, as they are not upregulated in infected flies [21]. It has been suggested that *CHKov1*, which contains a choline kinase domain, might make flies resistant to organophosphates by affecting choline metabolism in general or the target of organophosphate insecticides, acetylcholine esterase [20]. If this is the case, it is possible that it could be linked to the mechanism of virus resistance as Rhabdoviruses use acetylcholine receptors to enter cells [28].

Did the *Doc* insertion initially function as a defence against viruses or insecticides? Previous work has shown that there is extensive linkage disequilibrium between the *Doc* insertion and surrounding sites [20]. These observations, which we confirmed using a much larger dataset, provide compelling evidence for a partial selective sweep in which the *Doc* insertion has very recently increased in frequency. However, the number of sequence changes that have accumulated in the *Doc* element suggest that the insertion occurred approximately 90,000 years ago, which long predates the use of insecticides [20]. The most recent common ancestor of present-day sigma virus isolates existed roughly 2,000 years ago [17], and the infection may have been present in fly populations for much longer than this. Therefore, the *Doc* element would initially have only played a role in defending flies against viral infection, but these flies found themselves with an unexpected advantage once organophosphate insecticides were introduced.

The duplication of this region that resulted in the allele with the highest level of virus resistance has occurred very recently. There are only 2 sequence differences between our mapping lines in the 30 kB region surrounding the duplication, compared with over 550 polymorphisms among the DGRP lines. For this reason it is unsurprising that this highly resistant allele is still rare in the wild (although we have not tested flies from the population where this allele was first found). It is possible that given sufficient time this allele may replace the partially resistant allele that dominates today's populations.

Taken together, our results show that successive changes to the same genomic region have caused large shifts in the resistance of flies to the sigma virus. These mutations have all resulted in substantial structural changes to the genes involved, and the first of them has swept through populations under directional selection. This has not only increased the resistance of flies to viral infection, but it may also have pre-adapted flies to the introduction of insecticides in the middle of the last century.

## Materials and Methods

### Fly lines and crosses

A susceptible (22a) and resistant (OOP) fly line was provided by Didier Contamine. The third chromosome of OOP is derived from the Paris line [19] and carries both the resistant allele of the *ref(3)D* gene and an allele of a gene called *ref(3)V* which reduces the transmission of the virus through sperm [19]. The remaining chromosomes of OOP are from the susceptible Oregon R lab stock. Before attempting to map *ref(3)D* we first separated it from *ref(3)V* by crossing OOP and 22a. The F1 progeny were then crossed to *TM6B, Tb/Sb*, and the resulting *TM6B,Tb/*+ male progeny back-crossed to the balancer stock. These flies were then genotyped using molecular markers located at 92 cM and 94 cM on the standard genetic map. As these markers lie between *ref(3)D* and *ref(3)V*, this allowed us to identify a recombinant that carried the resistant allele of *ref(3)D* but not *ref(3)V*.

To map *ref(3)D* we created stocks that carried homozygous chromosomes that were recombinants between the resistant and susceptible chromosomes. We crossed the resistant stock to 22a, and crossed the F1 progeny to *TM6B,Tb/Sb*. Single male *TM6B,Tb/+* progeny were then crossed back to the balancer. A few days after setting up this cross the males were removed from the tube and genotyped using molecular markers at 80 cM and 92 cM, which flank the region thought to contain *ref(3)D* [19]. This allowed us to retain just the 21 genotypes that had recombined in this region. In the next generation we crossed sibling *TM6B,Tb/+* flies, and then selected for homozygous recombinants in the subsequent generation. Once we had mapped the gene to a smaller region (see below), we then repeated the experiment using different molecular markers to produce another 33 recombinants between 86 cM and 88 cM.

To select recombinants in even smaller regions we used phenotypic markers flanking the region of interest rather than molecular markers. First, we selected two lines, *w^1118^; P {GT1}BG02256* and *w^1118^;P {GT1}jigr1^BG00794^*, which carry *P*-elements flanking the region of interest. These elements both carried the mini-white gene, and flies that carry a single heterozygous element have lighter colored eyes than flies carrying two heterozygous elements [29]. This allowed us to cross them and select a 3^rd^ chromosome mapping line that carries both elements (2GT1). This was then crossed to a resistant 3^rd^ chromosome recombinant line (D2-6) generated in the experiment described above. Recombinants between 2GT1 and D2-6 were then generated as in the previous experiment, except that this time the 3^rd^ chromosome recombinant lines were balanced with *w^−^;TM3,Sb/H* and recombinants were detected from their eye color. Ten homozygous 3^rd^ chromosome recombinant lines were generated along with controls with either no recombination event or a recombination event outside the region of interest.

To generate recombinants at defined sites in the vicinity of the resistance gene we used *P*-element-induced male recombination [30]. Four different lines with transposable element insertions (*P*-elements) (Text S1) were used with a resistant line to generate recombinants via male induced recombination. The crossing scheme was kindly provided by Kevin Cook (Text S1) and *w^−^;TM3,Sb/H* was used to balance the lines. Non-recombinant lines with either the 3^rd^ chromosome derived from the susceptible *P*-element line or the resistant parental stock used in this cross (see Text S1) were generated as controls. All four transposable insertion lines contained the second allelic variant (Figure 4B) of *CHKov1* (data not shown).

### Genotyping flies from mapping crosses

DNA was extracted using either a protocol using Chelex resin (Sigma-Aldrich, St Louis) [31] or a Tissue Genomic DNA Kit (Metabion, Munich). Genotyping was done using microsatellites, indels, SNP specific primers or via sequencing (Table S1). To score length differences in indels and microsatellites, short PCR products were run on 2% agarose gels, while larger products were run on 1% agarose gels. PCR products for sequencing were cleaned up by incubating with the enzyme Exonuclease I and Shrimp Alkaline Phosphotase at 37°C for 1 hr, followed by a 15 min incubation at 72°C to deactivate the enzymes. The sequencing reaction consisted of 25 cycles of 95°C (30 sec), 50°C (20 sec) and 60°C (4 min) using BigDye reagents (ABI). Sequencing was carried out at either Source BioScience LifeSciences (Cambridge) or The GenePool (Edinburgh).

### Sigma virus

The Hap23 strain of the sigma virus [32] was extracted from an infected line of *D. melanogaster* (Om), and this extract was used in all assays except one. One hundred flies were ground in 1 ml of Ringer's solution, centrifuged at 13000 rpm for 30 seconds, and the supernatants from several replicate tubes mixed together. The viral extract was then separated into small aliquots and stored at −80°C. When this ran low, the same procedure was followed, this time using susceptible flies two weeks after they were injected with the previous stock of sigma virus. This new stock was tested on susceptible and resistant lines and then used in the 3^rd^ chromosome 2GT1 experiment.

### Measuring resistance

Female *D. melanogaster* were injected in the abdomen with sigma virus until slight extension of the proboscis was observed. They were then maintained on either Lewis media or apple juice-agar media. Flies were tipped onto new media two days after injection and then two more times before they were tested for infection. The flies were then exposed to 100% carbon dioxide for 15 minutes at 12°C on day 10 after injection (the first two recombinant assays) or day 14 (all subsequent assays). Flies were given 2 hours to recover from the carbon dioxide and then the number of dead or paralyzed individuals was counted as well as the total number of individuals in each vial. Four replicate vials each containing approximately 15 flies on average were used in each experiment except for the first recombinant assay with the third chromosome line (three replicates).

### Sequencing and genotyping candidate regions

DNA for sequencing was extracted using the kit described above. The majority of the 59.6 kb region on chromosome 3 that we had identified by mapping using recombinant lines (3R:21126075..21185688; release 5.31 of the *Drosophila* genome) was sequenced from both OOP and 22a (GenBank accession numbers JN247668–JN247669). Primer pairs were designed to amplify these regions in overlapping fragments (Table S1), and the sequencing was performed as described above. The sequencing of a small region involving the genes CHKov1 and CHKov2 was made more difficult by a complex rearrangement in which certain sequences had been duplicated. This region was therefore sequenced by designing PCR primers that amplified just single copies of the duplicated region.

Diagnostic PCR primers were designed to genotype flies for a *Doc* element insertion in *CHKov1* and a complex rearrangement involving *CHKov2*. The forward primer CHK2-8F (5′ GCAGCACGATCGTCAAATAG 3′) and the reverse primer CHK2-8R (5′ AATGCTTCAAAGGTTTTGTTGA 3′) were used to detect the absence of the insert near *CHKov2*. The forward primer CHK2-7F (5′ TCTTCTCATCTTCCGGGACT 3′) and the reverse primer FlipR (5′ GTAGTTACTGGACCACAAGTTGAAG 3′) were used to identify the presence of the 5′ end of the insertion near *CHKov2*. The forward primer CHK_F (5′ CTCTTGGCTCCAAACGTGAC 3′) and reverse primer CHK_R (5′ AAGGCAAACGACGCTCTT 3′) were used to detect the absence of the *Doc*1420 element in *CHKov1*. The forward primer *Doc*1420_F (5′ CTTGTTCACATTGTCGCTGAG 3′) was used with the reverse primer CHK_R to detect the presence of the *Doc*1420 element in *CHKov1*. The genotype of another resistance gene, *ref(2)P*, was scored using the PCR test described in [33].

### Quantitative RT-PCR

To examine the expression of candidate resistance genes and estimate viral titers we used quantitative rtPCR. Four biological replicates of 8 resistant and 11 susceptible recombinant lines were injected with sigma virus, and RNA was extracted from two of the replicates after 6 days and the other two replicates after 12 days (1 resistant line missing second 12-day replicate). From each biological replicate we extracted RNA from 10 individuals using Trizol (Invitrogen) following the manufacturer's instructions. RNA was reverse transcribed into cDNA using MMLV (Invitrogen) and random hexamer primers. Viral load was determined using quantitative PCR using SYBR Green and the forward primer DmelSV_F1 (5′ TTCAATTTTGTACGCGGAATC 3′) and reverse primer DmelSV_R1 (5′ TGATCAAACCGCTAGCTTCA 3′), which amplify a region of the viral genome spanning the L gene and 5′ trailer (and therefore amplify genomic RNA but not mRNA). Expression of CHKov1 was measured using the forward primer CHKoV1-qPCR-F1 (5′ GAACTCCGTGGGATCGACTA 3′) and reverse primer CHKoV1-qPCR-R2 (5′ CATGGGACAGGTGTTTGTCA 3′). These primers span the first intron of the gene, and amplify a region of the gene that is present in the truncated form of the gene (described below). Expression of CHKov2 was measured using the forward primer CHK2_3F (5′ CACCAAAAATCTCCGTGGTT 3′) and reverse primer qPCR_Chkov2_3_R (5′ TCGTTCTCATAAGCGACTATACATC 3′). Expression of *Actin 5C* was used as a control in all assays using the primers qActin5c_for2 (5′ GAGCGCGGTTACTCTTTCAC 3′) and qActin5c_rev2 (5′ AAGCCTCCATTCCCAAGAAC 3′). We performed three technical replicates of each PCR and used the mean of these in subsequent analyses.

### Drosophila Genetic Reference Panel

To test which naturally-occurring polymorphisms are associated with resistance we used the *Drosophila* Genetic Reference Panel, which is a panel of highly inbred fly lines from North America whose genomes have been sequenced (http://www.hgsc.bcm.tmc.edu/project-species-i-Drosophila_genRefPanel.hgsc). To measure the resistance of these lines, we injected 186 of the lines with the virus and tested them for infection 13 days later. In total we tested 11870 flies for infection, and on average 4 different replicate vials of each line containing an average of 16 flies were tested. As far as was possible, each replicate vial of each line was injected on a different day and on each day we used different combinations of lines.

### Analysis

R version 2.11.1 was used for statistical analyses. Our data from the infection experiments consists of numbers of infected and uninfected flies, which we treat as a binomial response in a generalized linear mixed model. The parameters of the model were estimated using the R library MCMCglmm [34], which uses Bayesian Markov chain Monte Carlo (MCMC) techniques. To test for an association between *Doc*1420 status and resistance to sigma virus we used the model:
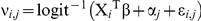
Where ν*_i,j_* is the probability of flies in vial *i* from line *j* being infected. β is a vector of the fixed effects of *ref(2)P* genotype and *Doc*1420 genotype, and X*_i_* ^T^ is a row vector relating the fixed effects to vial *i*. α*_j_* is a random effect of line *j*. The residual, ε*_i,j_*, includes over-dispersion due to unaccounted for heterogeneity between vials in the probability of infection. The estimated effect of *Doc*1420 on infection rates was back-transformed from logits into a proportion, and the number quoted in the text is based on estimates for lines that have the susceptible allele of *ref(2)P*. The 95% highest posterior density of the MCMC sample was used as an estimate of the credible intervals (C.I.) of parameters.

This Bayesian approach is computationally intensive and slow to implement, so when testing larger numbers of SNPs from the DGRP dataset for effects on resistance we used a maximum likelihood method. The model was essentially the same as that described above except the SNP in question was included as a fixed effect (and *Doc*1420 status was not always included). The model was fitted using the R function *lmer*, and the significance of the fixed effects was assessed using the Wald statistic. When sample sizes are small this can give anti-conservative results [35], but this should not be important in our analysis as common SNPs were found to be highly significant (see below).

For each fly line in which we measured viral titres or gene expression by quantitative RT-PCR, we first calculated Δ*Ct* as the difference between the cycle thresholds of the gene of interest and the endogenous control (*actin 5C*). The viral titre or gene expression in resistant flies relative to susceptible flies was calculated as 2^−ΔΔ*Ct*^, where ΔΔ*Ct* = Δ*Ct_resistant_*−Δ*Ct_susceptible_*, where Δ*Ct_resistant_* and Δ*Ct_susceptible_* are the means of the Δ*Ct* values of the resistant and susceptible lines. To assess whether these differences were statistically significant, we used a Wilcoxon Rank Sum Test to compare Δ*Ct* in the resistant lines and the susceptible lines. This calculation assumes that the PCR reactions are 100% efficient. To check whether this assumption is realistic we used a dilution series to calculate the PCR efficiency. Using this approach we found that the actin PCR is 103% efficient, the virus PCR is 101.5% efficient, the *CHKov1* PCR is 100.0% efficient and the *CHKov2* PCR is 102.5% efficient.

## Supporting Information

Table S1 List of primers used for genotyping markers and for sequencing 60 kb region.
(XLS)

Text S1 List of lines and crossing scheme used to generate *P*-element-induced male recombinant lines.
(DOC)
