# Supplementary material for: Successive Increases in the Resistance of Drosophila to Viral Infection through a Transposon Insertion Followed by a Duplication
Source: PLoS Genet. 2011 Oct 20;7(10):e1002337. doi: 10.1371/journal.pgen.1002337 (PMC3197678; doi:10.1371/journal.pgen.1002337)
Supplement: Text S1 — List of lines and crossing scheme used to generate P-element-induced male recombinant lines. (DOC) [file pgen.1002337.s002.doc]

**Methods S1**

Lines used:

D2-6 – resistant stock

*w*;TM3,Sb*/*H* – 3^rd^ chromosome balancer

*y^1^ w*; CyO, H* {*w^+mC^*=*P*Delta2-3}HoP2.1/*Bc^1^* – 2^nd^ chromosome balanced transposase

*st^1^ Sb^sbd-1^ e^s^ ro^1^ ca^1^* – mapping stock

*P*-element lines Genome location

P {EPgy2}CG10669^EY10583^ 3R:21158162

P {EPgy2}EY02779 3R:21155073

P {EPgy2}EY10184 3R:21129819

P {EPgy2}EY21769 3R:21128160

Crossing scheme part 1:

1G_0_: *st^1^ Sb^sbd-1^ e^s^ ro^1^ ca^1^* x resistant line

1G_1_: *st^1^ Sb^sbd-1^ e^s^ ro^1^ ca^1^* / resistant females *x st^1^ Sb^sbd-1^ e^s^ ro^1^ ca^1^* males

1G_2_: *w*;TM3,Sb / H* females x *st^1^ e^+^* {resistant} *ro^+^ ca^1^* males

1G_3_: *TM3 / st^1^* {resistant} *ca^1^* x *TM3 / st^1^* {resistant} *ca^1^*

1G_4_: *st^1^* {resistant} *ca^1^* stock

Crossing scheme part 2:

2G_0_: *y^1^w*;CyO*, Δ2-3 females x *w*;TM3,Sb / H* males

2G_1_: *st^1^* {resistant} *ca^1^* females x *w**; *CyO*, Δ2-3 / + ; *TM3,Sb* / + males

2G_2_: *P* {*w^+^*} insertion female x *CyO, Δ2-3 / + ; st^1^* {resistant} *ca^1^ / TM3,Sb* males

2G_3_*: st^1^ Sb^sbd-1^ e^s^ ro^1^ ca^1^* females x *CyO*, Δ2-3 / +; *st^1^* {resistant} *ca^1^* / *P*-insertion males

2G_4_: *w*; TM3, Sb / H* females x (recombinant *st^-^ ca^+^ or st^+^ ca*^-^) / *st^1^ Sb^sbd-1^ e^s^ ro^1^ ca^1^* males

2G_5_: *TM3, Sb* / (recombinant *st^-^ e^+^ ca^+^ or st^+^ e^+^ ca*^-^) x *TM3, Sb* / (recombinant *st^-^ e^+^ ca^+^ or st^+^ e^+^ ca^-^*)

2G_6_: recombinant stock
